# A rare neurological adverse event of palbociclib: a chronic inflammatory demyelinating polyneuropathy (CIDP) mimic

**DOI:** 10.1007/s10549-026-08081-6

**Published:** 2026-11-04

**Authors:** Joy Zeynoun, Madeline Evans, Kimberly Boldig, Haythem Ali

**Affiliations:** 1 Department of Neurology, Henry Ford Health, Detroit, MI USA; 2 Wayne State University School of Medicine, Detroit, MI USA; 3 Department of Hematology/Oncology, Henry Ford Health, Detroit, MI USA; 4 Michigan State University, East Lansing, MI USA

**Keywords:** Palbociclib, Breast Cancer, Immune mediated polyradiculoneuropathy

## Abstract

**Purpose:**

Hormone receptor-positive (HR+)/human epidermal growth factor receptor 2-negative (HER2–) breast cancer accounts for approximately 65–70% of all breast cancer cases. First-line treatment for metastatic HR+/HER2– disease typically includes endocrine therapy combined with a CDK4/6 inhibitor, achieving progression-free survival (PFS) of 24–33 months with preserved quality of life. Although CDK4/6 inhibitors are generally well tolerated, they are associated with hematologic and gastrointestinal toxicities. We describe a patient who developed features of chronic immune-mediate polyradiculoneuropathy associated with neurofascin antibodies following initiation of palbociclib.

**Methods:**

We conducted a detailed clinical review of a patient with metastatic HR+/HER2– breast cancer who developed progressive bilateral upper and lower extremity weakness after starting palbociclib. Clinical presentation, laboratory findings, imaging studies, electrodiagnostic testing, and treatment response were reviewed.

**Results:**

A 43-year old female with metastatic HR+/HER2- breast cancer developed asymmetric progressive weakness and sensory symptoms approximately two weeks after initiating palbociclib. Electrodiagnostic testing demonstrated a mixed axonal and demyelinating sensorimotor polyneuropathy meeting EAN/PNS criteria for an acquired inflammatory demyelinating polyradiculoneuropathy. Initial diagnostic considerations included acute inflammatory demyelinating polyradiculoneuropathy (AIDP) and multifocal CIDP (formerly termed multifocal acquired demyelinating sensory and motor neuropathy [MADSAM]). Despite initial improvement following treatment with IVIG, the patient experienced continued neurologic progression requiring plasmapheresis, repeat IVIG, high-dose corticosteroids, and rituximab. Subsequent testing demonstrated anti-neurofascin-155 and anti-neurofascin-186 antibody positivity. Longitudinal follow-up demonstrated significant functional recovery following rituximab therapy.

**Conclusion:**

This case suggests a possible association between palbociclib exposure and the development of chronic immune-mediate polyradiculoneuropathy associated with neurofascin antibodies. Clinicians should consider immune mediated neuropathies in patients receiving CDK4/6 inhibitor therapy who develop weakness, sensory deficits, and areflexia. Further investigation is warranted to better characterize potential immunologic effects of CDK4/6 inhibition and their relationship to autoimmune neurologic disease.

## Introduction

Breast cancer remains one of the most prevalent malignancies in the United States and is the most frequently diagnosed cancer among women, accounting for approximately 30% of new cancer diagnoses in females each year [1]. Consequently, the development of effective and well-tolerated therapeutic strategies is essential to improve patient outcomes and reduce disease burden.

Cyclin-dependent kinase 4/6 inhibitors (CDK4/6i), including palbociclib, ribociclib, and abemaciclib, have become a cornerstone of treatment for HR+/HER2- advanced or metastatic breast cancer [2, 3]. In combination with endocrine therapy, these agents have demonstrated significant improvements in both progression free survival and overall survival compared with endocrine therapy alone and are now standard first-line therapy for metastatic HR+/HER2- disease [2–4].

Oncologic care requires a careful balance between achieving effective tumor control and minimizing treatment-related toxicity. Although generally well tolerated, CDK4/6 inhibitors are associated with a variety of adverse effects, including neutropenia, fatigue, diarrhea, nausea, hepatotoxicity, and QT prolongation [2, 5]. Neurologic adverse events are uncommon and have largely been limited to headache, dizziness, peripheral neuropathy, and rare reports of posterior reversible encephalopathy syndrome (PRES) [5–7].

We report a patient who developed chronic immune-mediate polyradiculoneuropathy associated with neurofascin antibodies shortly after initiation of palbociclib therapy. To our knowledge, this represents the first reported case associated with palbociclib exposure.

## Case presentation

A 43-year-old right-handed woman with Stage IV infiltrating ductal carcinoma (IDC) of the right breast (cT4b, cN1, cM1, G3, ER+, PR+, HER2-) with known metastases to the brain, spine, and lungs was started on therapy with abemaciclib, letrozole, and leuprolide. Six months following initiation of this treatment regimen, abemaciclib was discontinued and palbociclib was initiated due to an acute kidney injury.

Approximately two weeks after initiating palbociclib, the patient developed numbness involving the fingertips and toes. Initial evaluation attributed these symptoms to borderline-low vitamin B12 levels (272 pg/mL), diabetes mellitus (HbA1c 6.5%), and a potential medication side effect. Methylmalonic acid and homocysteine levels were subsequently normal. Despite B12 supplementation, symptoms progressed. Weakness that initially presented in the left upper extremity progressed to involve the right upper extremity and both lower extremities. Palbociclib was discontinued four weeks following initiation due to worsening neurologic symptoms. No antecedent infectious illness, vaccination, respiratory infection, or gastrointestinal illness was identified.

The patient was hospitalized six weeks following palbociclib initiation due to progressive weakness and inability to ambulate. Neurologic examination demonstrated diffuse areflexia, distal sensory loss involving the hands and feet, and weakness that was more pronounced in the upper extremities (4/5 proximally and 3/5 distally). Cranial nerve examination was normal.

MRI of the brain and spine showed no intracranial metastases, leptomeningeal disease, or spinal cord compression. Stable osseous metastatic disease involving the thoracolumbar spine was present with nerve root compression at L5–S1 but no findings sufficient to explain the severity or distribution of symptoms. Cerebrospinal fluid analysis showed normal glucose, borderline elevated protein (52.9 mg/dL), mild pleocytosis (11 nucleated cells/µL), negative infectious studies, and no malignant cells on cytologic examination.

Electromyography and nerve conduction studies performed demonstrated a mixed sensory greater than motor large-fiber peripheral neuropathy with both axonal and demyelinating features affecting the bilateral upper extremities greater than the lower extremities. Diffuse denervation was present throughout the sampled right upper extremity and proximal lower extremity muscles. Findings fulfilled 2021 European Academy of Neurology/Peripheral Nerve Society (EAN/PNS) electrodiagnostic criteria for acquired inflammatory demyelinating polyradiculoneuropathy [8]. Given the asymmetric upper extremity onset and multifocal distribution, multifocal CIDP was considered in the differential diagnosis [9].

Repetitive nerve stimulation demonstrated an 11.3% decrement of the left spinal accessory nerve after exercise that was not reproduced in the ulnar nerve. Acetylcholine receptor antibodies and muscle-specific kinase antibodies were negative, making a primary neuromuscular junction disorder unlikely. Subsequent serologic testing demonstrated positivity for both anti-neurofascin-155 and anti-neurofascin-186 antibodies at a titer of 1:32 by immunofluorescence assay.

The patient initially received IVIG (0.4 g/kg/day for five days) with only limited improvement. Ongoing progression prompted treatment with plasmapheresis one month after her initial treatment. Despite these interventions, weakness worsened and progressed to severe quadriparesis requiring repeat hospitalization. Additional treatment included repeat plasmapheresis, high-dose intravenous methylprednisolone, repeat IVIG, and rituximab administered twice.

Following rituximab therapy, gradual neurologic improvement was observed. At subsequent neuromuscular follow-up, the patient reported substantial recovery of upper extremity strength and functional independence. She regained the ability to dress herself, tie her shoes, stand from a seated position, and ambulate short distances without a walker. Residual deficits included mild distal sensory loss involving the feet and persistent balance impairment requiring physical therapy.

## Discussion

At initial presentation, the differential diagnosis included AIDP, acute-onset CIDP, multifocal CIDP, paraneoplastic neuropathy, and autoimmune nodopathy. This clinical presentation overlapped substantially with AIDP, including rapidly progressive weakness, areflexia, sensory deficits, and inflammatory electrodiagnostic features. AIDP classically presents with progressive bilateral and relatively symmetric weakness, often ascending from the distal lower extremities, with reduced or absent deep tendon reflexes and progression reaching nadir within two to four weeks [10].

However, several findings argued against a typical AIDP presentation. Symptoms initially began asymmetrically in the upper extremities before progressing to involve the contralateral arm and lower extremities, an atypical pattern for AIDP, which more commonly presents with symmetric ascending weakness [10]. Furthermore, despite treatment with IVIG, the patient continued to deteriorate clinically beyond eight weeks, ultimately progressing to severe quadriparesis requiring plasmapheresis, repeat IVIG, high-dose corticosteroids, and rituximab. This prolonged disease course refractory to treatment suggested an underlying chronic immune-mediated neuropathy rather than a monophasic post-infectious process [10–12].

CIDP is an acquired immune-mediated neuropathy characterized by progressive or relapsing sensorimotor dysfunction extending beyond eight weeks and associated with peripheral nerve demyelination [10–12]. Although typical CIDP presents with symmetric proximal and distal weakness, numerous atypical variants have been described. One such variant, multifocal CIDP, also known as Lewis-Sumner syndrome, is characterized by asymmetric sensory and motor deficits with multifocal nerve involvement [9, 13, 14].

The patient’s initial asymmetric upper-extremity involvement was suggestive of multifocal CIDP. Previous studies have demonstrated that atypical CIDP variants frequently exhibit distinct electrophysiologic profiles and may differ in treatment response compared with typical multifocal CIDP [14]. Multifocal CIDP is thought to result from focal immune-mediated injury to peripheral nerves and may involve disruption of the blood-nerve barrier at sites of conduction block [13, 15]. Unlike AIDP, multifocal CIDP has not been consistently associated with antecedent infections or vaccinations and is generally considered part of a chronic inflammatory neuropathy spectrum [13].

Despite these multifocal CIDP -like features, definitive classification remained challenging. Over time, the patient’s disease evolved into severe symmetric quadriparesis with extensive disability, creating overlap among acute-onset CIDP, multifocal CIDP, and autoimmune nodopathy. This evolution highlights the diagnostic complexity frequently encountered in inflammatory neuropathies and underscores the importance of longitudinal clinical assessment [11, 12].

The subsequent identification of anti-neurofascin-155 and anti-neurofascin-186 antibodies shifted the diagnostic consideration toward autoimmune nodopathy. Autoimmune nodopathies are increasingly recognized as distinct disorders separate from classic CIDP and are characterized by antibodies directed against nodal and paranodal proteins involved in saltatory conduction [16]. Recognition of these disorders has significantly altered the diagnostic framework of inflammatory neuropathies, leading the 2021 EAN/PNS guidelines to classify autoimmune nodopathies as entities distinct from CIDP [17].

Pan-neurofascin antibodies, which recognize both NF155 and NF186, have been associated with a more severe phenotype that may include rapidly progressive sensorimotor neuropathy, cranial neuropathies, autonomic dysfunction, and respiratory compromise [19, 20]. Compared with patients with typical CIDP, individuals with autoimmune nodopathy frequently demonstrate more severe weakness, greater disability, and reduced responsiveness to IVIG therapy [16, 18, 19]. Conversely, increasing evidence suggests improved outcomes with B-cell-directed therapies such as rituximab. This pattern was reflected in our patient, who experienced continued neurologic progression despite IVIG and plasmapheresis but subsequently demonstrated substantial functional recovery following rituximab administration. Importantly, anti-NF155 autoimmune nodopathy is most strongly associated with antibodies of the IgG4 subclass, which were not assessed by the commercial antibody assay used in our patient, limiting further serologic characterization of the neurofascin antibodies detected [21].

Despite these findings, there are several features which argue against a classic presentation of anti-NF155 autoimmune nodopathy. This typically presents with distal-predominant, symmetric weakness, tremor, ataxia, and markedly elevated CSF protein [20]. Our patient developed asymmetric weakness, had only low-normal CSF protein, and lacked significant tremor or ataxia. Diffuse denervation on needle EMG and mild CSF pleocytosis are also atypical for autoimmune nodopathy, which characteristically shows electrophysiologic demyelinating features without prominent axonal injury and elevated CSF protein without pleocytosis [22]. Although anti-neurofascin antibodies support nodal/paranodal immune involvement, they are not entirely specific for autoimmune nodopathy as antibodies against neurofascin isoforms have been reported in patients with genetic, idiopathic, and other inflammatory neuropathies, and may arise secondarily in the setting of severe ongoing nerve injury [23]. Similarly, our patient lacked the fulminant presentation typical of pan-neurofascin antibody-associated disease, which includes cranial neuropathies, autonomic dysfunction, and respiratory failure [24]. Taken together, these findings suggest that although neurofascin antibodies likely reflect an immune-mediated nodal process, the patient’s phenotype does not fully conform to classic anti-NF155 or pan-neurofascin autoimmune nodopathy, highlighting the diagnostic overlap among inflammatory neuropathies.

Alternative etiologies were carefully considered. Neuroimaging demonstrated no evidence of leptomeningeal disease, spinal cord compression, or other structural abnormalities capable of explaining the patient’s symptoms. Cerebrospinal fluid cytology was negative for malignant cells, arguing against carcinomatous meningitis or direct neoplastic involvement. No antecedent infectious illness, vaccination, or other established trigger for AIDP was identified. Borderline vitamin B12 deficiency was not supported by normal methylmalonic acid and homocysteine levels, and diabetic neuropathy would not be expected to produce the rapid progression, diffuse areflexia, quadriparesis, and inflammatory electrodiagnostic abnormalities observed in this case. Similarly, negative acetylcholine receptor and MuSK antibody testing made a primary neuromuscular junction disorder unlikely.

A paraneoplastic process cannot be entirely excluded given the known association between malignancy and immune-mediated neuropathies, however, several features argue against this etiology. Paraneoplastic neurologic syndromes are rare and are typically associated with antibodies directed against intracellular neuronal antigens such as anti-Hu, CRMP5, amphiphysin, and anti-Yo [25]. In breast cancer, paraneoplastic neurologic syndromes most commonly manifest as cerebellar degeneration, myelopathy, or myopathy rather than inflammatory demyelinating neuropathy [26]. Furthermore, the 2021 PNS-Care criteria state that neuropathies meeting diagnostic criteria for CIDP should not be considered paraneoplastic in the absence of a high-risk onconeural antibody [27]. In our patient, anti-Hu (ANNA-1) and CRMP5 antibodies were negative. Although not all paraneoplastic antibodies were evaluated, the negative results for these high-risk onconeural antibodies make a paraneoplastic etiology less likely.

The temporal relationship between palbociclib initiation and symptom onset raises concern for a treatment-associated immune-mediated neuropathy. Symptoms developed approximately two weeks after starting palbociclib and continued to progress despite discontinuation of therapy. Although causality cannot be established from a single case, the chronology is notable and biologically plausible given emerging evidence regarding the immunomodulatory effects of CDK4/6 inhibition.

Preclinical studies have demonstrated that palbociclib suppresses proliferation of activated T lymphocytes through G0/G1 cell-cycle arrest while largely preserving cytotoxic effector function [28]. Importantly, regulatory T cells appear particularly susceptible to CDK4/6 inhibition. Selective reduction of regulatory T-cell populations may shift the balance between immune tolerance and immune activation, potentially creating conditions favorable for the emergence of autoimmunity [28].

Clinical studies have further supported these observations. The ORACLE-RIPA trial demonstrated differential immune modulation among CDK4/6 inhibitors, while the NeoRHEA study demonstrated reductions in regulatory T cells and myeloid-derived suppressor cells during treatment with palbociclib-based therapy [4, 29]. These findings suggest that CDK4/6 inhibition can substantially alter the immune microenvironment in vivo.

Additional experimental studies suggest that CDK4/6 inhibition also alters innate immune signaling pathways, further supporting the concept that these agents exert immunomodulatory effects beyond tumor cell-cycle regulation [30]. Although these observations were made in glioma models, they further support the concept that CDK4/6 inhibitors exert broad immunologic effects extending beyond malignant cells.

The detection of both anti-neurofascin-155 and anti-neurofascin-186 antibodies suggests involvement of an immune-mediated process directed against nodal and paranodal proteins, although interpretation should be made in the context of the patient’s atypical clinical features and absence of IgG subclass characterization. Recognition of autoimmune nodopathies remains critical because these disorders often exhibit distinct treatment responses and prognostic implications compared with classic CIDP [16–20].

Although peripheral neuropathy has been reported in pharmacovigilance analyses of CDK4/6 inhibitors, chronic immune-mediate polyradiculoneuropathy associated with neurofascin antibodies has not previously been described [6]. The rarity of this presentation limits definitive conclusions regarding causality. Nevertheless, several observations support a potential association. Neurologic symptoms developed shortly after treatment initiation of palbociclib, extensive evaluation failed to identify an alternative etiology, objective evidence of immune-mediated neuropathy with anti-neurofascin antibody positivity was demonstrated, and emerging evidence of immune modulation by CDK4/6 inhibitors provides biologic plausibility for such an association.

To our knowledge, this represents the first reported case of chronic immune-mediate polyradiculoneuropathy associated with neurofascin antibodies temporally associated with palbociclib exposure. While causation cannot be proven from a single observation, this case expands the spectrum of potential neurologic adverse events associated with CDK4/6 inhibitors and highlights the need for continued post-marketing surveillance. Additional studies are needed to determine whether CDK4/6 inhibition can trigger, unmask, or accelerate autoimmune neurologic disease in susceptible individuals.

## Conclusion

We report a case of atypical chronic immune-mediate polyradiculoneuropathy associated with neurofascin antibodies developing shortly after initiation of palbociclib in a patient with metastatic HR+/HER2 − breast cancer. Although the initial presentation shared features with AIDP and multifocal CIDP, progression beyond eight weeks, treatment refractoriness, anti-neurofascin antibody positivity, and substantial improvement following rituximab therapy suggest an underlying immune-mediated neuropathy. While the detection of anti-neurofascin antibodies raised suspicion for autoimmune nodopathy, the patient’s clinical phenotype was not entirely consistent with classic anti-NF155 or pan-neurofascin antibody-associated disease, highlighting the diagnostic overlap among inflammatory neuropathies.

This case expands the spectrum of potential neurologic toxicities associated with CDK4/6 inhibitors and highlights the importance of considering autoimmune nodopathy in patients who develop progressive weakness, sensory loss, and areflexia during treatment. Recognition of these disorders is clinically important because they may respond differently to immunotherapy than classic CIDP. Further investigation is warranted to clarify the relationship between CDK4/6 inhibition and immune-mediated neurologic disease.

## Data Availability

No datasets were generated or analysed during the current study.
